# Fabrication of Carbon Disulfide Added Colloidal Gold Colorimetric Sensor for the Rapid and On-Site Detection of Biogenic Amines

**DOI:** 10.3390/s21051738

**Published:** 2021-03-03

**Authors:** Namhyeok Choi, Bumjun Park, Min Ji Lee, Reddicherla Umapathi, Seo Yeong Oh, Youngjin Cho, Yun Suk Huh

**Affiliations:** 1 NanoBio High-Tech Materials Research Center, Department of Biological Sciences and Bioengineering, Inha University, 100 Inha-ro, Incheon 22212, Korea; cnhc2482@gmail.com (N.C.); jbrianpark@gmail.com (B.P.); minji3989@gmail.com (M.J.L.); umapathi4u@gmail.com (R.U.); 2 Research Group of Consumer Safety, Korea Food Research Institute, 245 Nongsaengmyeong-ro, Iseo-myeon, Wanju-gun, Jeollabuk-do 55365, Korea; seyeongoh@kfri.re.kr

**Keywords:** biogenic amines, on-site detection, carbon disulfide, colloidal gold, colorimetric sensor

## Abstract

Meat is often wasted due to the perceived concerns of its shelf life and preservation. Specifically, in meat formation, biogenic amines (BAs) are the major agents to spoil them. Herein, we have developed a carbon disulfide (CS_2_) added colloidal gold nanoparticles-based colorimetric sensor for the rapid and on-site detection of biogenic amines. Transmission electron microscopy is used to observe the morphological changes in colloidal gold nanoparticles and aggregation behavior of CS_2_ added to the colloidal gold nanoparticles’ solution. Raman spectroscopic analysis is further used to characterize the peaks of CS_2_, Cad and CS_2_-Cad molecules. Absorption spectroscopy is used to estimate the colorimetric differences and diffuse reflectance spectra of the samples. The sensing analysis is performed systematically in the presence and absence of CS_2_. CS_2_ added colloidal gold nanoparticles colorimetric sensor detected the BAs with a limit of detection (LOD) value of 50.00 µM. Furthermore, the developed sensor has shown an LOD of 50.00 µM for the detection of multiple BAs at a single time. The observed differences in the colorimetric and absorption signals indicate that the structure of BAs is converted to the dithiocarbamate (DTC)-BA molecule, due to the chemical reactions between the amine groups of BAs and CS_2_. Significantly, the developed colorimetric sensor offers distinct features such as facile fabrication approach, on-site sensing strategy, rapid analysis, visual detection, cost-effective, possibility of mass production, availability to detect multiple BAs at a single time and appreciable sensitivity. The developed sensor can be effectively used as a promising and alternative on-site tool for the estimation of BAs.

## 1. Introduction

Over the past few years, consumption of high-quality meat and meat products has been increasing steeply on a large scale [1,2]. However, decomposition and deterioration of meat usually happen due to the action of microorganisms and various offensive odor substances, such as hydrogen sulfide (H_2_S), methane (CH_4_), sulfurous compounds, ammonia (NH_3_) and biogenic amines (BAs). All these substances are released from the amino acids (AAs), as they are disassembled from the proteins present inside the foods [3,4,5,6]. Among these food deteriorations, deterioration caused by the BAs is in large numbers. Chiefly BAs are produced when the food or food products were exposed to oxygen for long periods, allowing the foods to be available for the microbial oxidative hydro decarboxylation of the AAs [7]. Principally, BAs are nitrogenous biomolecules with very low molar mass bearing functional groups as the organic bases. BAs can be classified into two different cases, in which the first case is based on the number of amine groups in BAs, which are monoamines, diamines and polyamines. The second case is based on the chemical structure of BAs, which are aliphatic compounds such as cadaverine (Cad), putrescine (Put), spermine (Spm), and spermidine (Spd) and aromatic compounds such as tyramine (Trm) and β-phenylethylamine, and heterocyclic compounds such as histamine (Him), tryptamine and serotonin [8,9].

A low concentration of BAs produced from various types of food is essential to take action on gastric acid output and thermoregulation of the body; on the other hand, a high concentration of BAs is toxic to the body, which causes food poisoning or converts them to strong carcinogens [10]. Especially, Cad and Put become highly toxic as they easily form nitrosamines by reacting with the nitrous acid, whereas Trm and Him will affect the blood vessels and nervous system, which ultimately causes hypertension and headaches [11,12]. Therefore, continuous monitoring of BAs detection is required, especially in high-protein products such as meat products, which can easily be exposed to BAs.

Currently, analysis of BAs in various food products is performed through various conventional instruments, such as high-performance liquid chromatography (HPLC), gas chromatography (GC) and capillary electrophoresis. These instruments can potentially measure BAs qualitatively and quantitatively with very high sensitivity and selectivity, but the price of the measurements is very expensive, and professional skills are required for the analysis, in which these instruments demand long-time consumption from the extraction, preprocessing and analysis [13,14,15,16,17]. Real-time polymerase chain reaction (RT-PCR), another method to detect BAs, can analyze a large number of samples very quickly and accurately. Moreover, with this technique, it is possible to estimate additional production of BAs during the distribution of the channels. On the other hand, RT-PCR is not an adequate technique to monitor BAs during the product production time, because this is not a direct quantitative analysis method [18]. BAs can also be exploited by enzyme-linked immunosorbent assay (ELISA), which quantitatively measures a combination degree of antigens and antibodies using enzyme activation after polymerizing the enzymes on the antigen or antibody. This method analyzes samples quickly with a large amount, but it is not possible to measure the total amount of BAs inside the food qualitatively and quantitatively [19].

Another strategic method for the detection of BAs is surface plasmon resonance (SPR), which utilizes the charge density oscillation effect that occurred on the surface of the thin metal film. Chemical and physical changes that occurred on the fixed samples of the thin metal film can be measured through the resonance angle or the reflectance produced by the reducing reflexibility. Moreover, surface plasma wave conversion can happen during the absorption of light energy on the surface of the thin metal film [20,21,22]. Based on these characteristics, SPR based sensors and biosensors have several advantages, as they can quantitatively analyze different biochemical and biological effects with high sensitivity, and without labeled substances [23]. However, SPR instruments are associated with the high price requirement of a large number of samples for the fixation on the sensor chips [24]. Thus, this method is also not suitable for the rapid and on-site detection of BAs.

From these inferences, we can ascertain that traditional BA sensing techniques are time-consuming, and they are not suitable for the rapid and on-site detection of BAs. Thus, rapid, precise, accurate, consumer-friendly, cost-effective and on-site detection of the food quality is of utmost necessity for the safety and wellbeing of mankind globally. Over the past few years, significant attention has been made to maintain the quality and freshness of meat by developing novel detection techniques. Among the various types of sensing strategies, colorimetric sensing has been designated as a potential strategy for the efficient detection of BAs in meat and meat products.

In this regard, colorimetric sensors based on the localized surface plasmon resonance (LSPR) effect of colloidal golds are developed, and various types of BAs are further determined. Colloidal golds were synthesized through a reduction reaction of gold ions with citric acids as stabilizers, and the detection of BAs was possible with particle aggregation effects. Combination possibility between different types of separate BAs and colloidal gold was determined and reactivity between a mixture of BAs and colloidal gold was further confirmed. Therefore, plasma absorption-based colorimetric sensors are expected as next generation sensors in food nutrition fields to effectively detect various types of BAs under different conditions.

## 2. Materials and Methods

### 2.1. Materials

Gold (III) chloride trihydrate (HAuCl_4_∙3H_2_O; ≥99.9%), carbon disulfide (CS_2_; ≥99.9%), cadaverine (Cad; C_5_H_14_N_2_, ≥97.0%), 1.4-diaminobutane (putrescine (Put); C_4_H_12_N_2_, ≥99.9%), histamine (Him; C_5_H_9_N_3_, ≥97.0%), tyramine (Trm; C_8_H_11_NO, ≥99.0%), L-Histidine (His; C_6_H_9_N_3_O_2_, ≥99.0%), L-lysine (Lys; C_6_H_14_N_2_O_2_, ≥98.0%), L-Arginine (Arg; C_6_H_14_N_4_O_2_, ≥98.0%) and L-Glutamine (Glu; C_5_H_10_N_2_O_3_, ≥99.0%) were purchased from Sigma-Aldrich Chemicals, USA. Trisodium citrate dihydrate (Na_3_Ctr; Na_3_C_6_H_5_O_7_) was purchased from Kanto chemicals, Japan. Ultrapure water with a resistivity of 18.2 MΩ.cm was used for all the experimental procures and analysis.

### 2.2. Synthesis of Colloidal Gold Solution

In total, 150.0 mL of 2.2 mM Na_3_Ctr solution was heated to 100.0 °C under vigorous stirring for 15 min. Later, 1.0 mL of 25.0 mM HAuCl_4_∙3H_2_O solution was added slowly and stirred for 30 min for the formation of colloidal gold seeds. Further, 53.0 mL of distilled water and 2.0 mL of 60.0 mM Na_3_Ctr solution were added to the solution and stirred for another 20 min. Finally, 1.0 mL of 25.0 mM HAuCl_4_∙3H_2_O solution was added slowly and stirred for 30 min for the synthesis of the colloidal gold nanoparticles solution with uniform shape.

### 2.3. Functionalization of Colloidal Gold Nanoparticles-Based Colorimetric Sensor with Carbon Disulfide

Considering the molar ratio of the particles, CS_2_ and Cad were taken in a 2:1 ratio and stirred for 10 min. Raman spectroscopic analyses were performed to confirm the peaks of the CS_2_ and Cad molecules and to identify the formation of dithiocarbamate(DTC)-Cad. To interpret the interactions and for the functionalization of CS_2_ and colloidal gold solution, CS_2_ was initially diluted with double the concentration of the target molecule. Further, 500.0 µL of the diluted solution was mixed with an equal amount of colloidal gold with an optical density value of 1, then the colloidal mixture was stirred for 10 min, this functionalization resulted in the fabrication of colloidal gold nanoparticles-based colorimetric sensor.

### 2.4. Colorimetric Sensing of Biogenic Amines in Distilled Water

For the colorimetric sensing of BAs in distilled water, colloidal gold nanoparticles’ solution was mixed with and without CS_2_. BA samples viz Cad, Put, Him and Trm were prepared in three different types of mixture samples, in which sample 1 consists of Cad, Put and Trm, sample 2 consists of Cad, Put and Him, and sample 3 consists of Cad, Him and Trm. All these samples were prepared in the concentration range of 1.0 to 1000.0 µM. For the activation of the sensor, 500.0 µL of each sensing solution and the prepared BAs samples were mixed and kept under stirring for 10 min, later the absorbance spectrum of the samples was measured to ascertain the aggregation behavior of the colloidal gold.

### 2.5. Characterization

Transmission electron microscopy (TEM; CM200, Philips, Amsterdam, The Netherlands) images were used to study the morphological changes in the colloidal gold solution. Raman spectroscopic analyses were performed using a Raman spectrometer (FEX, NOST, Seongnam City, Korea) in the range of 300–2000 cm^−1^. Colorimetric differences and UV-vis diffuse reflectance spectra of the samples were measured using ultraviolet-visible (UV-vis) spectrophotometer (V-770, JASCO, Tokyo, Japan) in the range of 300–800 nm.

## 3. Results

### 3.1. Fabrication of Colloidal Gold-Based Colorimetric Sensors

**Figure 1:**
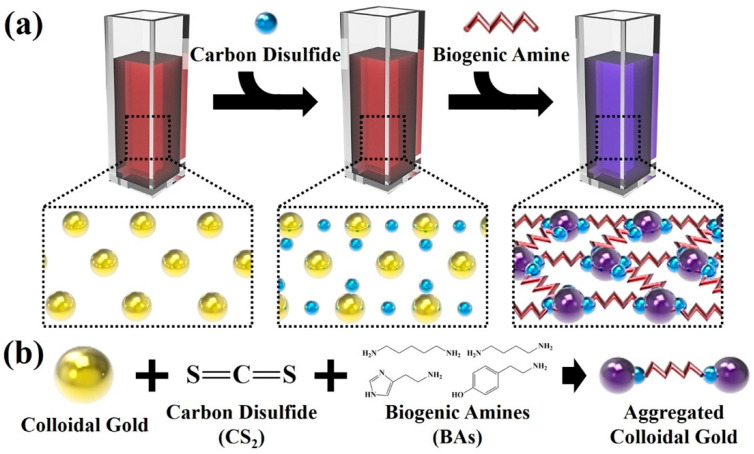
Schematic illustration of (**a**) the colorimetric detection of biogenic amines based on the simple mixing of carbon disulfide and gold nanoparticles and (**b**) aggregation of colloidal gold nanoparticles solution with the addition of carbon disulfide and biogenic amines.

Colorimetric sensors could simply be fabricated by the reaction of colloidal gold solution. Schematic illustration of BA detection through colorimetry based on the simple mixing of CS_2_ and colloidal gold nanoparticles was described in Figure 1a. Colorimetric sensing of BAs was easily performed by the simple reaction of colloidal gold solution. Specifically, the addition of BAs to the colloidal gold solution and CS_2_ mixture resulted in the occurring of interactions between the colloidal gold nanoparticles, CS_2_ and BAs. All these molecules, with their associated molecular interactions, lead to the aggregation of colloidal gold nanoparticle solution. Under the visible light, the colloidal gold solution itself emitted strong red light, due to the LSPR effect. Absorption spectra of colloidal gold particles showed a red shift due to the aggregation of the particles or the peak shift effect when the surrounding refraction rates of chemical or biological particles were attached to the surface of the gold particles [25]. These phenomenological mechanisms brought the changes in the absorbance spectrum further, where they were further differentiated from the chemical groups attached to the particles [26]. With the addition of CS_2_ to the colloidal gold solution, characteristic changes were not observed in the color, representing that molecular interactions of the CS_2_ and the colloidal gold solution did not undergo any changes and both the molecules were properly mixed. Finally, with the addition of the selected BAs to the colloidal gold/CS_2_ colorimetric sensor, the color of the sensing solution has been changed to blue, which indicated the aggregation mechanism of the colloidal gold solution. Figure 1b schematically depicts the aggregation of colloidal gold/CS_2_ solution upon the addition of CS_2_ and BAs. The reactions occurring in the colorimetric sensor could be ascertained based on the binding groups of the BAs. CS_2_ could not directly interfere with the colloidal gold solution, whereas colloidal gold/CS_2_ solution interfered with each other with the help of an amine group of BAs, which further lead to the formation of DTC. Generally, the binding group of BAs change from the amine group to DTC through the reaction with CS_2_. To lead the aggregation of colloidal gold nanoparticles for colorimetric sensing of BAs, two DTC molecules are required as CS_2_ could not directly bind to the colloidal gold nanoparticles. With the formation of DTC, the length of the molecules is increased, and the molecular interaction changes from electrostatic interaction between carboxyl and amine group to chemisorption between DTC and surface of colloidal gold nanoparticles [27]. All these molecules aggregated the colloidal solution [28,29]. The cohesion and the observed molecular changes of colloidal gold nanoparticles, CS_2_ and BAs were further analyzed through obtained TEM analysis. The TEM image of the colloidal gold/CS_2_ solution (Figure S1a) signified the cohesion between colloidal gold nanoparticles and CS_2_. The average size of the synthesized nanoparticles was 13.49 ± 0.74 nm. The TEM image of colloidal gold/CS_2_ with BAs solution (Figure S1b) depicted the molecular changes and aggregation of colloidal gold/CS_2_ solution upon the addition of BAs.

### 3.2. Reactivity between Carbon Disulfide and Colloidal Gold Solution

**Figure 2:**
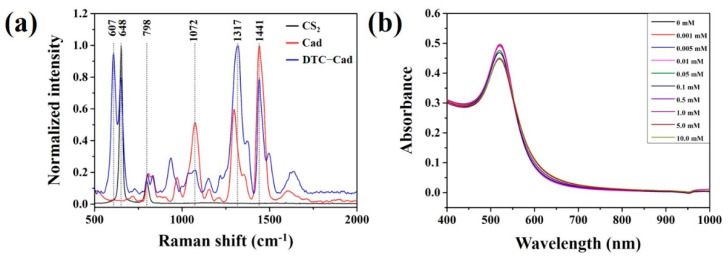
(**a**) Raman spectra of CS_2_, Cad and DTC-Cad, and (**b**) absorption spectra of DTC-Cad with various concentrations of CS_2_.

The colloidal gold/CS_2_ solution reacted with the amine groups of BAs and resulted in the formation of DTC. Rapid field analysis with the developed sensor was made possible in 10 min, as the BAs composed of DTC directly coupled with the colloidal gold nanoparticles. Interactions and molecular reactions between CS_2_ and BAs were verified through the Raman spectrum under the fingerprint frequencies as shown in Figure 2a. It was evident that the CS_2_ molecule showed the Fermi doublet at 651 cm^−1^ and 798 cm^−1^ owing to the C=S interaction, whereas the Cad showed the peaks at 1072 cm^−1^, 1317 cm^−1^, and 1441 cm^−1^, corresponding to C-C stretching, C-N stretching and CH_2_ scissoring, respectively [30,31]. The peak at 607 cm^−1^ represented the formation of DTC-Cad, which further indicated the interactions between CS_2_ and Cad, owing to the C=S interaction and occurrence of chemical structural changes.

Further, to get deeper insights on the DTC-Cad (CS_2_ and colloidal gold), UV-vis spectroscopy analysis was performed by adding various concentrations of CS_2_ to the DTC-Cad (Figure 2b). While increasing the concentration of CS_2_, the absorbance peak at the 500–550 nm wavelength range was slightly decreasing, but the significantly appreciable difference in the maximum wavelength was not identified at the 525 nm. This depicted that significant and characteristic reactions were not occurring in the colloidal gold solution with increasing the concentration of CS_2_. The observed reactions and molecular interactions observed between colloidal gold/CS_2_ and BAs signified that the developed fabricated sensor could be efficiently used for the detection of BAs with various concentration.

### 3.3. Colorimetric Sensing of Biogenic Amines through the Developed Colloidal Gold Nanoparticles Based Sensors

**Figure 3:**
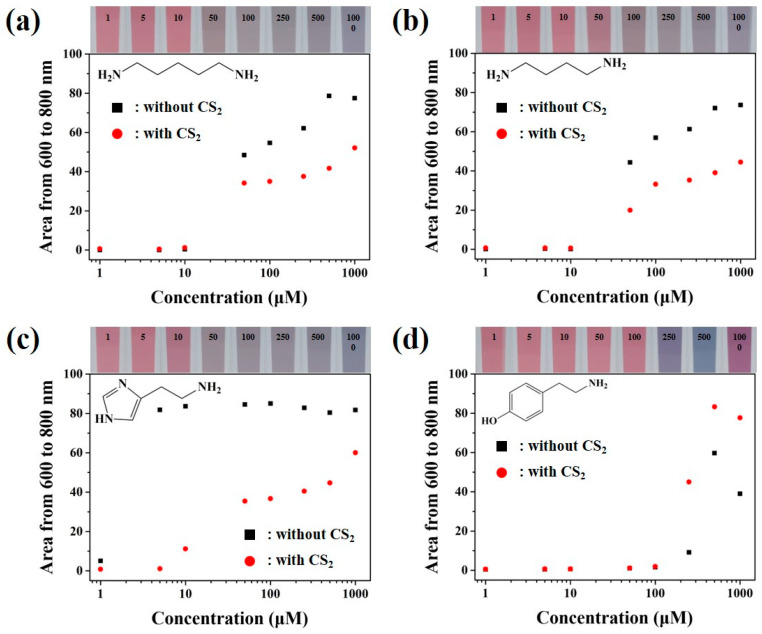
Absorption peaks of BA with various concentrations (**a**) Cad, (**b**) Put, (**c**) Him and (**d**) Trm with visual analysis of colorimetric sensors in the presence of CS_2_ for the detection of BA.

Reactivity studies between colloidal gold nanoparticles based colorimetric sensor and BAs such as Cad, Put, Him and Trm in the presence and absence of CS_2_ were performed to analyze the detection efficiency of the developed colorimetric sensor. The obtained absorption peaks of various concentrations of BAs detected using colloidal gold nanoparticles-based sensor was depicted in Figure 3. Representative visual analysis results of the colloidal gold nanoparticles-based colorimetric sensor and BAs in the presence and absence of CS_2_ were portrayed in Figure 3 inlets and Figure S2. Electrical repulsive forces of the negatively charged colloidal gold nanoparticles resulted in the good dispersion of the solution in the water. BAs molecules coupling with the colloidal gold particles were gradually increased due to the possible interactions between the side chains of the amine groups of BAs and gold nanoparticles. These reactions resulted in the formation of additional repulsive forces in the well-dispersed BAs molecules and coherence attractions between the colloidal gold solution and amine aliphatic groups of the BAs [32]. Therefore, with the addition of the respective specific concentration of BAs to the colloidal gold solution, repulsive and coherent forces between colloidal gold nanoparticles were perturbed as more amine aliphatic groups of the BAs interacted, and coupling interactions between the particles and the BAs were more favorable, resulting in the color change of colloidal gold solution by inducing particle aggregation.

In the case of colorimetric detection analysis of Cad in the presence and absence of CS_2_, significant changes in the color were not observed, which were evident from Figure 3a. In the absence of CS_2_ the active section of the solution concentration was ranged from 25.00 to 500.00 µM with the colorimetric signals ranging from 21.03 to 78.63, whereas in the presence of CS_2_, an active section of the solution concentration ranged from 25.00 to 1000.00 µM with colorimetric signals of 25.57 to 52.10. The obtained limit of detection (LOD) value was found to be 25.00 µM regardless of CS_2_. The observed differences in the colorimetric and absorption signals indicated that the structure of Cad was converted to the DTC-Cad molecule due to the chemical reactions between the amine groups of Cad and CS_2_. The formation of DTC-Cad resulted in the increase of the length of molecules and molecular interaction changes of the Cad binding group.

Figure 3b depicted the colorimetric detection analysis of Put. In the absence of CS_2_, Put showed the active section of the solution concentration between 50.00 and 500.00 µM with the colorimetric signals ranging from 44.37 to 72.03, whereas in the presence of CS_2_, an active section of the solution concentration ranged from 50.00 to 1000.00 µM with colorimetric signals ranging from 19.94 to 44.47. The LOD value for both the colloidal gold solutions was almost equal to 50.00 µM regardless of CS_2_. The representative colorimetric detection result of Put was similar to the Cad results. These similar phenomenologically resulted Cad and Put could be ascertained based on their chemical structures, as both the Put and Cad possess similar chemical structures. Put efficiently converted to DTC-Put, leading to the binding group transition. The colorimetric signals of Put were decreased when compared to Cad, due to the difference in the molecular length.

In the presence of Him, colorimetric changes of the colloidal gold solution in the presence and absence of CS_2_ were found to be apparent and distinctive, and the observed changes can be observed from the Figure 3c. In the absence of CS_2_, the LOD value was found to be 5.00 µM; in this solution activation section was not clear, which depicted the dramatic increase in the colorimetric signals and peaks from 1.00 and 5.00 µM. This type of phenomenon was due to the relationship between the structure of Him and the colorimetric reaction process. Precisely, in Him two amine groups were combined with the colloidal gold suspension and attached to the imidazole ring [33]. These structural changes optimized the interactions between Him and colloidal gold particles; further, these transformations resulted in the formation of more colloidal gold suspensions and a less activated amount of Him molecules by blocking the additional bindings between the molecules. However, in the case of colorimetric detection analysis of Him in the presence and absence of CS_2_, the LOD was increased to 10.00 µM with the expansion in the activation sites from 10.00 to 1000.00 µM with the colorimetric signals in the range from 11.17 to 60.02, which is quite similar to Cad and Put. This could be ascertained based on steric hindrance and the influence of the imidazole ring [34]. As both the steric hindrance and influence of the imidazole ring are decreasing with increasing the length of the molecules when the CS_2_ was coupled with the amine group of His, this process can be the reason for getting a similar type of reaction to Cad and Put.

Finally, the detection analysis of Trm was performed in the presence and absence of CS_2_, and the obtained findings were displayed in Figure 3d. Compared to the three BAs (Cad, Put and Him), Trm was structured with monoamines along with the characteristics of π-π interactions of the benzene ring and molecular interactions with colloidal gold would be induced like diamines [35]. However, when compared to other BAs, signals are largely reduced to 10 min due to the π-π interactions of the gold nanoparticles. When Trm was reacted with colloidal gold nanoparticles in the absence of CS_2_, LOD was found to be 250.00 µM and the colorimetric signal was increased from 250.00 to 500.00 µM. Nevertheless, the signal values were increased exceptionally at the same LOD value with the existence of CS_2_, this type of tendency could be due to the possibility of rapid π-π interactions and due to the increase of polarization differences in the benzene ring along with the structural change of amines through the CS_2_.

Changes in the plasmon spectrum of colloidal gold nanoparticles-based colorimetric sensors in the presence and absence of CS_2_ for the detection of BAs were confirmed through the absorption spectra analysis, the obtained spectra findings were illustrated in Figures S3 and S4.

### 3.4. Colorimetric Sensing of Mixed Biogenic Amines through Colloidal Gold Nanoparticles Based Sensors

**Figure 4:**
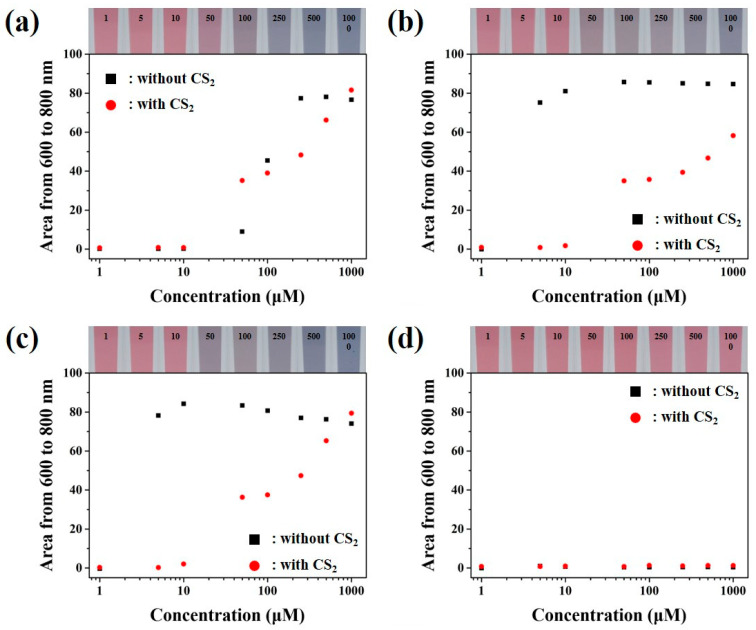
Absorption peaks of mixed BAs with various concentrations, (**a**) Cad, Put, Trm, (**b**) Cad, Put, Him, and (**c**) Cad, Him, Trm. (**d**) Adsorption peaks of multiple free amino acids (Arg, Lys, His and Glu) with various concentrations with visual analysis of colorimetric sensors in the presence of CS_2_ for the detection of mixed BAs.

The content of BAs generated by the decomposition of meat was gradually increasing based on the degree of decay concept, but the composition of BAs in the decomposed meat was changing either by the meat or with the changes in the environmental distribution [6,36]. Therefore, colorimetric sensors detecting only one type of BAs were not suitable to analyze the freshness of meat, and it was not appropriate for measuring the BAs index. In this regard, fabrication of the colorimetric sensors capable of detecting multiple BAs at a single take was of utmost importance and they were indeed required. In this regard, detecting the multiple BAs by using the developed colorimetric sensor was further researched. For the mixed BAs detection, the BAs (Cad, Put, Tyr and His) were prepared in three different types of mixture samples. The experimental reactivity and absorption peaks between mixed BAs samples and colloidal golds were analyzed systematically and the observed results were depicted in Figure 4.

In the absence of CS_2_, Cad, Put and Trm sample set showed LOD of 50.00 µM with signals of 8.95, whereas in the presence of CS_2_, signals showed a huge difference of up to 35.17 at the same limit of detection value (Figure 4a). In the absence of CS_2_, Cad, Put and Him mixture sample signals were shown at 75.16, at a LOD value of 5.00 µM, on the other hand, in the presence of CS_2_, signals were observed at 35.02, at a LOD value of 50.00 µM (Figure 4b). Similarly, in the absence of CS_2_, Cad, Him and Trm mixture sample set showed a LOD of 5.00 µM with the signals at 78.24, whereas in the presence of CS_2_, LOD value was varied to 50.00 µM with a lower signal of 37.52 (Figure 4c). As can be seen from Figure 3c, in the absence of CS_2_ the sensitivity of Him was lower than the other BAs, which could be due to the unclear or indefinite sensing behavior to detect Him. Whereas in the presence of CS_2_ the colloidal gold reactivity was equalized with all the BAs, in which the fabrication of the distinguishable colorimetric sensors was possible with the various concentration of BAs mixture samples. To get deeper insights on the developed colorimetric sensor, the mixture of free amino acids such as His, Lys, Arg and Glu were executed for the analysis (Figure 4d). The obtained results indicated that no significant reactivity was observed with the colloidal gold solution upon the addition of the amino acids. Moreover, as shown in Figure S8, the colloidal gold solution did not make any significant reactivity with the addition of small peptides such as glutathione (Glut) and carnosine (Carn). Finally, when colloidal gold solution was reacted to all different chemicals including amino acids, small peptides and BAs, the signals were shown to be high, indicating that colloidal gold nanoparticles were selectively reacted with BAs without interference of amino acids and small peptides. This is attributed to the interactions between the amine and carboxyl groups connected to the α-carbon chains of the free amino acids, which interfered with the combination of colloidal gold nanoparticles through the negative charges of the carboxyl group. The visual analysis results from the colloidal gold solution and BAs mixture were portrayed in Figure S5 and the UV-Vis/NIR absorbance spectrum graphs were shown in Figures S6 and S7.

## 4. Conclusions

In summary, a CS_2_ added colloidal gold nanoparticles-based colorimetric sensor was efficiently fabricated for the rapid and on-site detection of BAs. In the presence of CS_2,_ the formation of DTC with the BAs was confirmed through Raman and absorption spectroscopy analysis. The observed differences in the colorimetric and absorption signals indicated that the structure of BAs was converted to the DTC-BAs molecule, due to the chemical reactions between the amine groups of BAs and CS_2_. The formation of DTC-BAs resulted in the increase of the length of molecules and molecular interaction changes of the BAs binding group. All these interactions lead to changes in the aggregation of colloidal gold nanoparticles. Various types of reactivity interactions were observed during the detection of BAs with the developed colorimetric sensors in the presence of CS_2_. In the absence of CS_2_ sensitivity of Him was increased gradually, in which this phenomenological change made the detection a little difficult. However, LOD values of BAs were equalized to 50.00 µM with similar signaling to the colorimetric sensors with CS_2_ molecules. Furthermore, the fabricated sensor was employed to detect multiple BAs in a single run time.

Compared to recently published reports (Table S1), the fabricated colorimetric sensor has the following notable advantages:

(i) The fabrication of the sensor is very simple.
(ii) The developed colorimetric sensor detected the BAs within 10 min.
(iii) The developed sensor can be efficiently used for the on-site analysis of BAs.
(iv) The colorimetric sensor can be easily manufactured on the laboratory scale.
(v) Mass production of the colorimetric sensor will be highly possible at the industrial scale.
(vi) Notably, the developed sensor detects the multiple BAs at a single time.

The developed sensor will be useful to detect BAs in various types of foods, particularly to detect the freshness of meat and meat products. Further, we anticipate that the developed colloidal gold/CS_2_-based colorimetric sensor will bring novel insights and advancements for the fabrication of colorimetric sensors to detect various types of food pathogens.

## Data Availability

No new data were created or analyzed in this study. Data sharing is not applicable to this article.

## Supplementary Materials

The following are available online at https://www.mdpi.com/1424-8220/21/5/1738/s1, Figure S1: TEM images of colloidal gold nanoparticles, Figure S2: Visual analysis of colloidal gold nanoparticles based colorimetric sensor for the detection of BAs in the absence of CS_2_, Figure S3: Changes in the plasmon spectrum of colloidal gold nanoparticles based colorimetric sensor for the detection of BAs in the absence of CS_2_, Figure S4: Changes in the plasmon spectrum of colloidal gold nanoparticles based colorimetric sensor for the detection of BAs in the presence of CS_2_, Figure S5: Visual analysis of colloidal gold nanoparticles based colorimetric sensor in the absence of CS_2_ for the detection of mixed BAs, Figure S6: Changes in the plasmon spectrum of colloidal gold based colorimetric sensor in the absence of CS_2_ for the detection of mixed BAs, Figure S7: Changes in the plasmon spectrum of colloidal gold based colorimetric sensor in the presence of CS_2_ for the detection of mixed BAs, Figure S8: Selectivity of colorimet-ric sensor with various molecules, Table S1: Comparison of various methods for detection of BAs.
