# Supplementary material for: Fabrication of Carbon Disulfide Added Colloidal Gold Colorimetric Sensor for the Rapid and On-Site Detection of Biogenic Amines
Source: Sensors (Basel). 2021 Mar 3;21(5):1738. doi: 10.3390/s21051738 (PMC7959309; doi:10.3390/s21051738)
Supplement: Supplementary file 1 [file sensors-21-01738-s001.pdf]

Fabrication of a Carbon Disulfide added Colloidal Gold

Colorimetric Sensor for the Rapid and On-site Detection of

Biogenic Amines

Namhyeok Choi[1,‡], Bumjun Park[1,‡], Min Ji Lee[1], Reddicherla Umapathi[1], Seo Yeong Oh[2], Youngjin Cho[2,*], Yun Suk Huh[1,*]

[1] *NanoBio High-Tech Materials Research Center, Department of Biological Engineering, Inha University, 100 Inha-ro, Incheon, 22212, Republic of Korea*

[2] *Research Group of Consumer Safety, Korea Food Research Institute, 245 Nongsaengmyeong-ro, Iseomyeon, Wanju-gun, Jeollabuk-do 55365, Republic of Korea*

‡ These authors contributed equally to this work.

* Corresponding authors

E-mail address: yjcho74@kfri.re.kr (Y. Cho), yunsuk.huh@inha.ac.kr (Y.S. Huh)

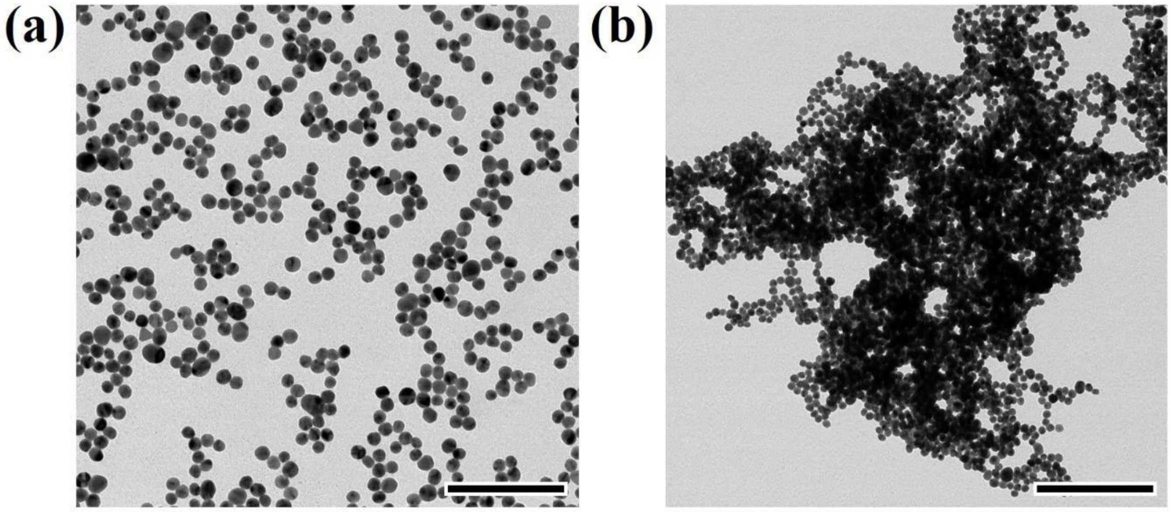

Figure S1. TEM images of colloidal gold nanoparticles **(a)** CS_2, and **(b)** mixture of CS_2 and BAs. Scale bars are 100.0 nm.

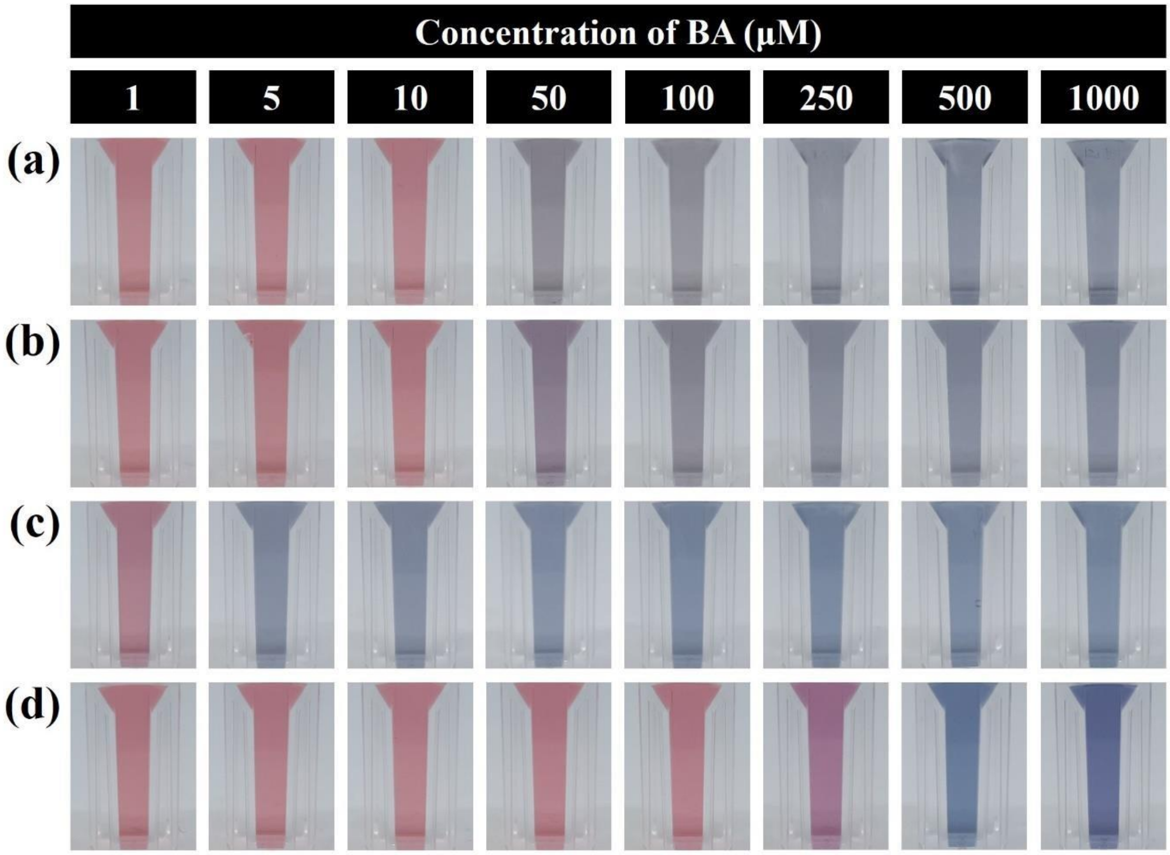

Figure S2. Visual analysis of colloidal gold nanoparticles-based colorimetric sensor for the detection of BAs **(a)** Cad, **(b)** Put, **(c)** Him, and **(d)** Trm, in the absence of CS_2.

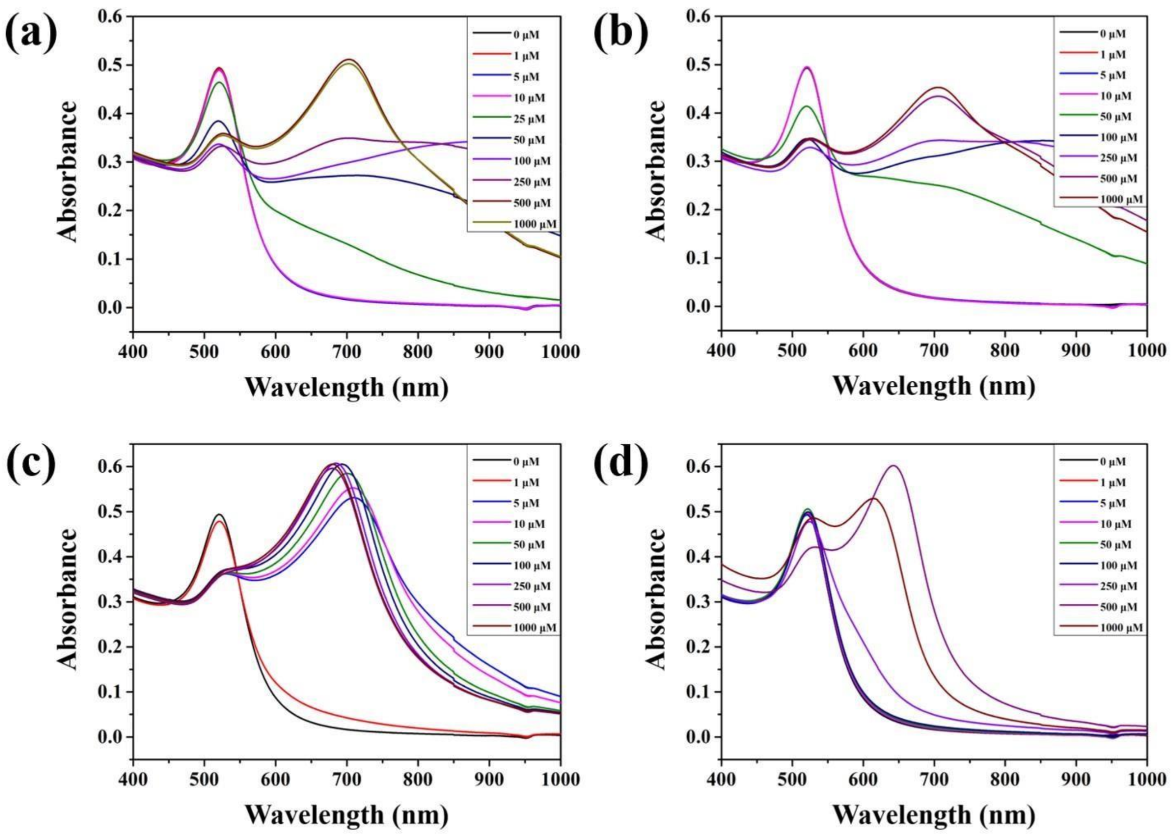

Figure S3. Changes in the plasmon spectrum of colloidal gold nanoparticles based colorimetric sensor for the detection of BAs **(a)** Cad, **(b)** Put, **(c)** Him, and **(d)** Trm, in the absence of CS_2.

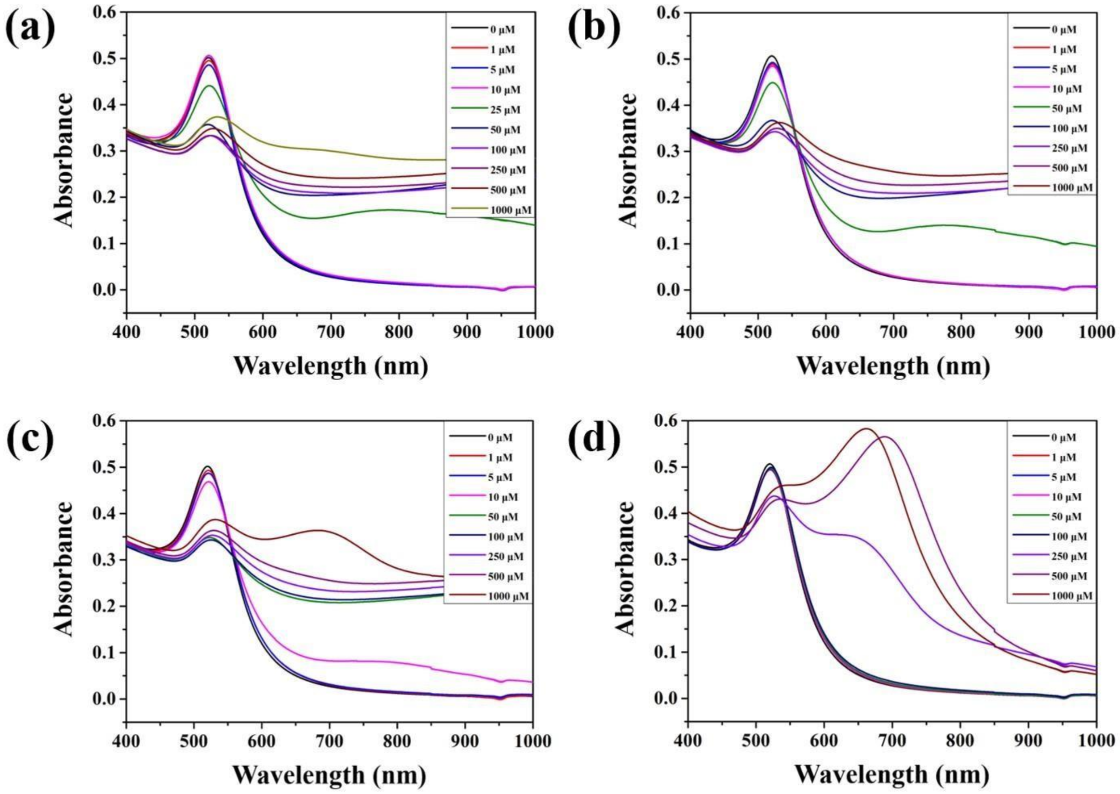

Figure S4. Changes in the plasmon spectrum of colloidal gold nanoparticles based colorimetric sensor for the detection of BAs **(a)** Cad, **(b)** Put, **(c)** Him, and **(d)** Trm, in the presence of CS_2.

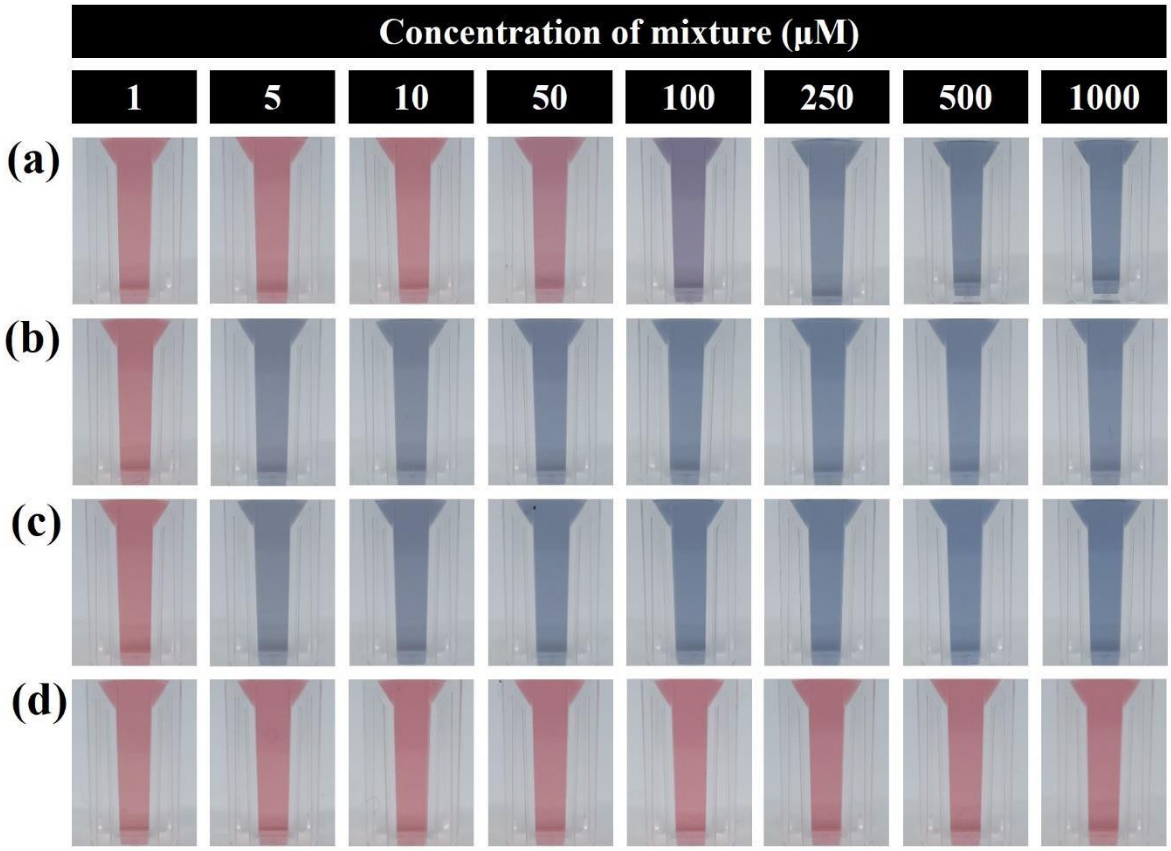

Figure S5. Visual analysis of colloidal gold nanoparticles based colorimetric sensor in the absence of CS_2 for the detection of mixed BAs; **(a)** Cad, Put, Trm, **(b)** Cad, Put, Him and **(c)**

Cad, Him, Trm; and **(d)** Visual analysis of the sensors reacted with different free amino acids (Arg, Lys, His and Glu) with various concentration.

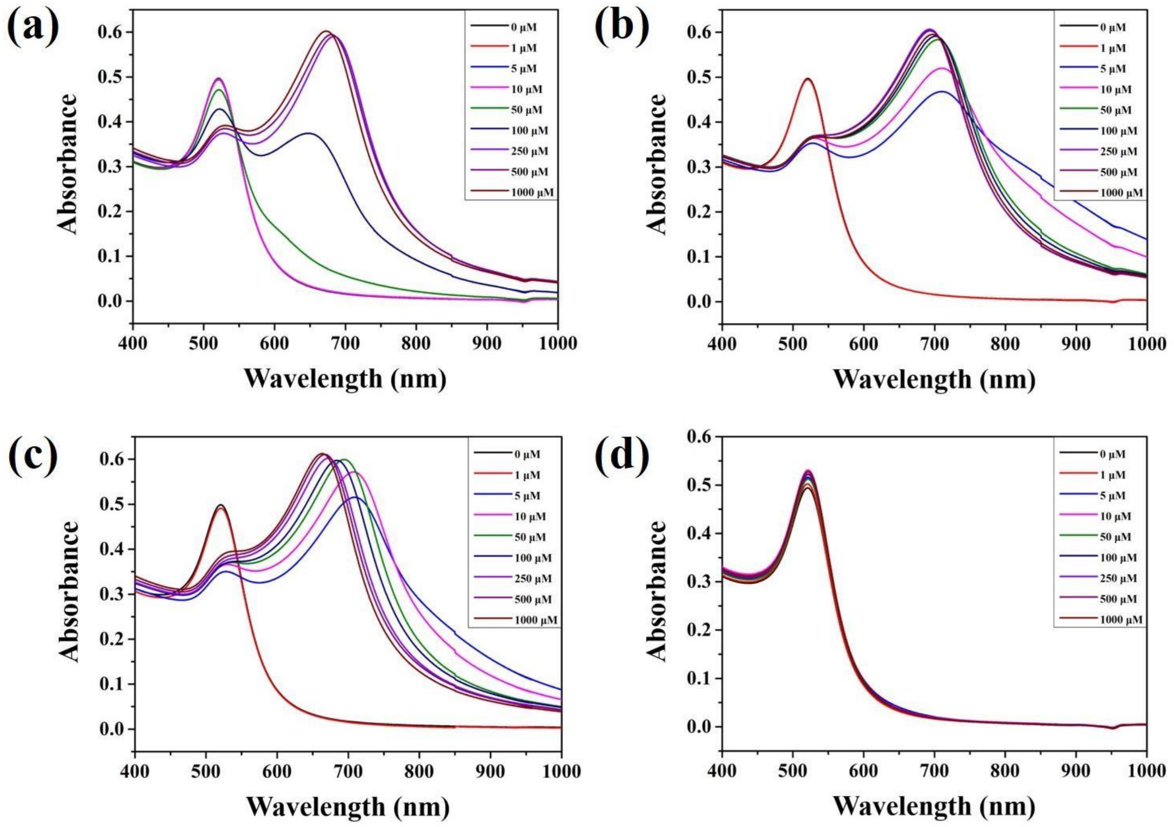

Figure S6. Change in the plasmon spectrum of colloidal gold based colorimetric sensor in the absence of CS_2, for the detection of mixed BAs **(a)** Cad, Put, Trm, **(b)** Cad, Put, Him and **(c)**

Cad, Him, Trm. **(d)** The change of plasmon spectrum with different free amino acids (Arg, Lys, His and Glu) under various concentrations.

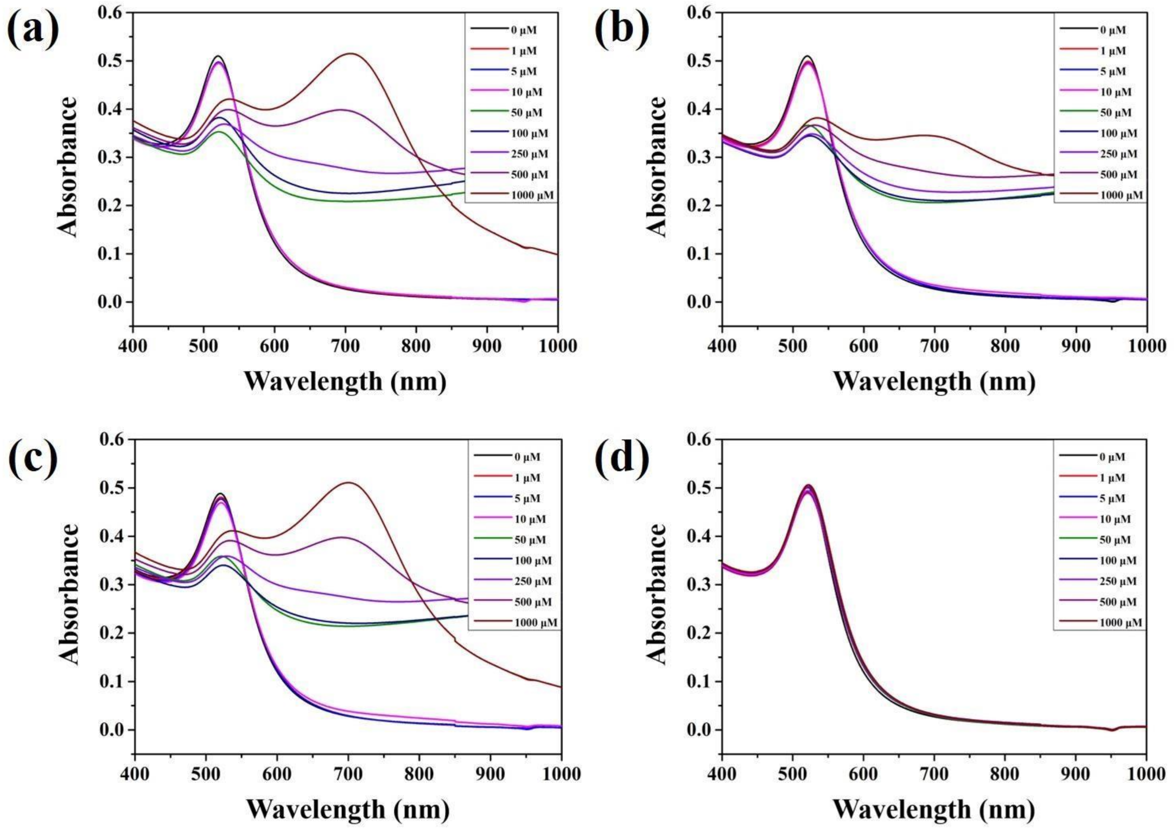

Figure S7. Changes in the plasmon spectrum of colloidal gold based colorimetric sensor in the presence of CS_2, for the detection of mixed BAs **(a)** Cad, Put, Trm, **(b)** Cad, Put, Him and **(c)**

Cad, Him, Trm. **(d)** The change of plasmon spectrum with multiple free amino acids (Arg, Lys, His and Glu) with various concentration.

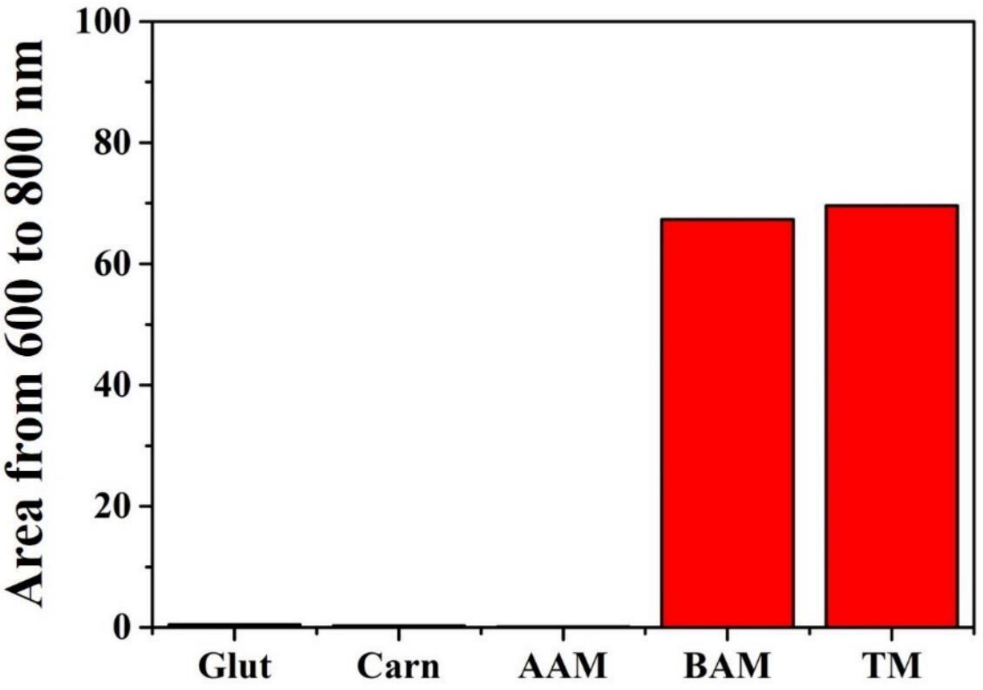

Figure S8. Selectivity of colorimetric sensor with various molecules such as small peptides (glutathione; Glut, and carnosine; Carn), amino acids (AAM; Arg, Lys, His, Glu), BAs (BAM; Cad, Put, Trm, Him) and total mixtures of small peptides, amino acids and BAs (TM).

Table S1. Comparison of various methods for detection of BAs.

BA Mixtures (Cad, Put, Trm, Him)	Analytical technique	LOD (μM)	LOQ (μM)	Ref.
Cad, Put, Trm	HPLC	5.0-30.0	10.0-60.0	[1]
Cad, Put, Trm, Him	HPLC	30.0-300.0	100.0-1000.0	[2]
Him	ELISA	100.0	400.0	[3]

Him	SPR	0.18	-	[4]
Cad, Put, Trm, Him	Colorimetric detection	50.0	100.0	This Work

References

1. Lázaro, C. A.; Conte-Júnior, C. A.; Cunha, F. L.; Mársico, E. T.; Mano, S. B.; Franco, R. M., Validation of an HPLC methodology for the identification and quantification of biogenic amines in chicken meat. *Food Anal. Methods* **2013,** 6, (4), 1024-1032.

2. Sirocchi, V.; Caprioli, G.; Ricciutelli, M.; Vittori, S.; Sagratini, G., Simultaneous determination of ten underivatized biogenic amines in meat by liquid chromatographytandem mass spectrometry (HPLC-MS/MS). *J. Mass Spectrom.* **2014,** 49, (9), 819-825.

3. Muscarella, M.; Magro, S. L.; Campaniello, M.; Armentano, A.; Stacchini, P., Survey of histamine levels in fresh fish and fish products collected in Puglia (Italy) by ELISA and HPLC with fluorimetric detection. *Food Control* **2013,** 31, (1), 211-217.

4. Lapenna, A.; Dell'Aglio, M.; Palazzo, G.; Mallardi, A., "Naked" gold nanoparticles as colorimetric reporters for biogenic amine detection. *Colloid Surf. A-Physcicochem. Eng.*
Asp. **2020,** 600, 124903.
